# Understanding quality of life in Danish women with metastatic breast cancer undergoing multiple treatments

**DOI:** 10.2340/1651-226X.2025.42446

**Published:** 2025-02-23

**Authors:** Helle Pappot, Annasofie Jørgensen, Anna Hincheli Bjørum, Christina Bøgh Jakobsen, Camilla Uhre Jørgensen, Beverley Lim Høeg, Pernille Bidstrup, Ann Knop, Line Bentsen

**Affiliations:** a Department of Oncology, Copenhagen University Hospital, Rigshospitalet, Copenhagen, Denmark; b Institute of Clinical Medicine, University of Copenhagen, Copenhagen, Denmark; c Psychological Aspects of Cancer, Danish Cancer Institute, Copenhagen, Denmark

**Keywords:** Metastatic survivorship, patient reported outcome, breast cancer, more treatment lines

## Abstract

**Background:**

Women with metastatic breast cancer (mBC) may experience several symptoms exacerbated by successive treatments. There is however, a lack of knowledge of the most important symptoms and how these may affect daily life function. This study aims to elucidate the quality of life (QoL), including both symptoms and daily life functions, among mBC women undergoing varied treatments.

**Methodology:**

We conducted a cross-sectional electronic questionnaire study enrolling mBC women (≥ stage III) receiving medical cancer treatment through September–December 2023. QoL, symptoms, and daily life function were measured using the European Organization for Research and Treatment of Cancer (EORTC) core questionnaire (QLQ-C30) and the breast cancer module (BR45). Health-related quality of life (HRQoL), defined by the EORTC, covers the subjective perceptions of the positive and negative aspects of cancer patients’ symptoms, including physical, emotional, social, and cognitive functions. We examined associations between QoL, treatment line and therapy types, and estimated odds ratios (ORs) and confidence intervals (CIs).

**Results:**

Of 359 eligible participants, 111 responded (30.9%). At study commencement, 90.9% of the participants received at least one type of systemic treatment, with 16.2% undergoing chemotherapy, 61.3% anti-hormonal treatment, and 66.6% targeted cancer treatment. QLQ-C30 sum scores were highest in women receiving anti-hormonal treatment (80.7, interquartile range [IQR]: 17.6), followed by targeted cancer treatment (78.8, IQR: 18.4), and lowest with chemotherapy (77.1, IQR: 24.8). Quality of life decreased with subsequent treatment lines (first line: 80.3, IQR: 20.7, fourth line: 67.4, IQR: 11.3). No significant differences were found in the functions or in the individual symptoms according to monotherapy type.

**Interpretation:**

Women with mBC experience a substantial symptom burden and reduced functioning, and their QoL differs with successive lines of treatment. This underlines that women living with mBC need support and effective symptom management to maintain QoL.

## Introduction

Every year, approximately 4,700 Danish women are diagnosed with breast cancer, with around 5% of these women presenting with primary metastatic disease (de novo) and a further 10% to 30% experiencing a systemic relapse within 10 years of their initial breast cancer diagnosis [1, 2]. The median overall survival (OS) of metastatic breast cancer (mBC) is around 3 years, and the 5-year OS is approximately 30% [1–3]. Present treatment options for mBC comprise both anti-hormonal treatment, chemotherapy, and targeted treatments, including modern immune checkpoint inhibitors (ICI) given as monotherapy or in combinations, sequential as the disease progresses [4, 5]. Each of these treatments is accompanied by a variety of possible side effects some of which are exacerbated by drug combinations [6] and not all reversible, leaving the women with the risk of an increasing late effects burden with increasing treatment lines [7]. A recent review by Ionescu et al. 2024, has described how women with mBC have gathered numerous symptoms and side-effects from a lived life with breast cancer comprising both consequences of prior surgery and radiation as well as late effects such as organ failure, for example cardiomyopathy, and symptoms based on metastasis, for example fractures due to bone metastasis [8]. Further, the women are presented with ongoing challenges, often exacerbated by the successive treatments, such as mBC’s impact on physical, cognitive, and functional functioning as well as body image and fatigue [9].

Together, these late effects contribute to lowered health-related quality of life (HRQoL). HRQoL, as defined by the European Organization for Research and Treatment of Cancer (EORTC), covers the subjective perceptions of cancer patients’ symptoms’ positive and negative aspects, including physical, emotional, social, and cognitive functions [10]. Recent data from more than 5,000 women have demonstrated how quality of life (QoL) measured by the EORTC QoL core questionnaire, EORTC QLQ-C30, was worse in women with mBC, having lower functioning scores and prevalence of more symptoms compared to women with early breast cancer (eBC) [11]. Furthermore, women with mBC may also experience breast cancer-specific challenges. To measure and follow such challenges, a breast cancer-specific QoL questionnaire has existed for nearly 30 years, the BR23 [12]. However, in the understanding of a changing treatment landscape for breast cancer patients, including targeted therapies, this questionnaire was recently enlarged to comprise 45 items, BR45 [13], now also covering in more detail sexual functioning. As the population of women living long-term with mBC will continue to grow due to improved treatment and survival, helping this population to sustain QoL will become increasingly important [14, 15].

A key challenge in supporting women with mBC is that it is difficult to predict their disease trajectory [16]. Treatment options used to be considered binary with either curative or palliative intent. However, with targeted treatment and immune therapies, the trajectories are getting much more difficult to predict [16], and thus, providing supportive care to alleviate the burden of symptoms is increasingly challenging. Thus, the introduction of new treatments combined with prolonged survival for women with mBC calls for a better understanding of metastatic survivorship [15, 17]. This study aimed, for the first time in a Danish context, to describe the QoL outcomes, including symptoms and daily life functions among mBC women according to different treatment modalities, and to present these findings to normative data.

## Material and methods

### Study population and recruitment

We conducted a cross-sectional questionnaire study, and between 01 October and 31 December 2023, we invited all eligible patients defined as women ≥ 18 years referred to or currently in ongoing treatment for mBC (≥ stage III) at the Department of Oncology, Copenhagen University Hospital, Rigshospitalet, Denmark. According to Danish Breast Cancer Group (DBCG), stage III and stage IV treatment is similar [18]. There were no further inclusion or exclusion criteria. All patients were asked to provide written informed consent prior to participation. All patients were invited in writing through the Danish E-boks, a communication platform including an electronic mailbox, where citizens in Denmark receive digital communication from public institutions, including the health care systems [19]. The study was approved by the authorities (P-2023-14404), and the STROBE guidelines were followed to ensure the proper reporting of the findings [20, 21].

### Data collection

The patients were asked to fill out an electronic questionnaire using the web-based platform REDCap [22]. At non-response, a reminder was sent by e-mail 21 days following the initial approach. Patients were asked to provide information on their civil status, children, living situation and occupational status.

### Quality of life

We measured symptoms, daily life function, and overall QoL using the EORTC [23] QLQ-C30 [24]. The questionnaire consists of five functional scales (physical, role, emotional, cognitive, and social functioning), nine symptom scales (fatigue, nausea and vomiting, pain, dyspnoea, insomnia, appetite loss, constipation, diarrhoea, and financial difficulties), and a global health status/QoL scale. All scales and single items are scored from 0 to 100. High scores indicate a high functioning or global health status for the functional scales and global health status. For the symptom scales, high scores indicate a high symptom severity. A sum score was established based on all sub-scales except financial difficulties [24]. For many countries, normative data on the QLQ-C30 are available for both genders, such as the recent English update from the United Kingdom [25]. However, the most recent Danish reference values are not separated by gender [26], but normative data from 2014 are available for Danish women [27].

### Breast cancer specific quality of life

We used QLQ-BR45 for measuring both breast cancer-related symptoms and toxicity. However, this questionnaire is not exhaustive concerning toxicity [13]. In this study, we defined treatment-related toxicity as every symptom from cancer-related therapy experienced by the patient. The QLQ-BR45 includes two multi-item scales: a target symptom scale and a satisfaction scale. The target symptom scale is divided into three subscales: endocrine therapy, endocrine sexual, and skin/mucosa scale. When our study was initiated, phase IV testing of the QLQ-BR45 was ongoing. The results of this test have led to the removal of three items. Thus, the most recent version, unavailable for our study, is the QLQ-BR42 [23]. The scoring principles for QLQ-BR45 are comparable to QLQ-C30 except for three scales: sexual enjoyment, sexual functioning, and breast satisfaction. These scales have an inverse correlation with function; thus, a high score indicates impaired function.

### Disease and treatment characteristics

Two medical doctors (CBJ and CUJ) collected disease and treatment characteristics from electronic medical records. These comprised age, years since diagnosis, hormone-receptor status, HER2-receptor status, and previous and present treatments. When responding to the questionnaire, treatment groups were defined as anti-hormonal/chemotherapy or targeted monotherapy (Table 1). We did not stratify the study population based on hormone- or HER2-receptor status due to the limitation of small sample sizes.

**Table 1:** Participants were stratified into treatment groups based on whether they were treated with antihormonal therapy/chemotherapy or targeted monotherapy.

Anti-hormonal therapy	Chemotherapy	Targeted monotherapy
Tamoxifen Letrozol Anastrozole Exemestan Fulvestrant Megestrolacetat	Docetaxel Paclitaxel Epirubricin Capecitabine Vinorelbine Gemcitabine Carboplatin-Gemcitabine Cyklophosphamid, Methotrexate, 5-Fluorouracil (CMF) Eribulin	Traztuzumab Pertuzumab Atelizumab Traztuzumab emtansine (TDM-1) Palbociclib Ribociclib Abemaciclib

### Statistical analyses

Descriptive analyses included medians (as data was not normally distributed) with interquartile range (IQR), and floor and ceiling effects. Floor and ceiling effects were calculated for the scales to assess the extent of minimum and maximum response levels. The floor effect indicates the proportion of respondents who scored the lowest possible value on a scale (0 points). The ceiling effect indicates the proportion of respondents who achieved the highest possible score (100 points). We included means to allow visual comparisons with normative data for EORTC QLQ-C30. We examined associations between clinical and sociodemographic factors, treatment line and therapy type, and QoL outcomes (below versus above median) using odds ratios (ORs). Mann-Whitney and Kruskal-Wallis tests were used for comparison between groups, and *p*-values < 0.05 were considered significant.

## Results

**Figure 1:**
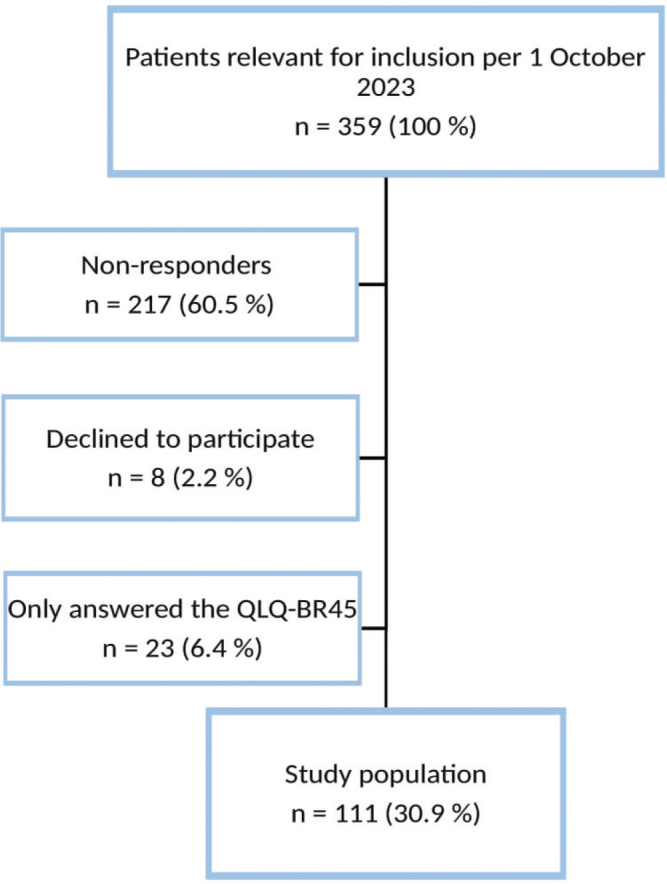
Flow-chart showing patient inclusion and reasons for incomplete data.

Out of 359 eligible participants, 111 responded (30.9%). The flowchart for the inclusion procedure is shown in Figure 1. Because of a technical error, women who had the questionnaires resend after a reminder only received QLQ-BR45; because of this, data from these 23 women were excluded from the analyses. The women had a mean age of 64.3 years and were at a mean of 11.3 years after the first diagnosis of breast cancer (Table 2). While 89.2% of the participants had estrogen-positive mBC, 24.3% had HER2-positive mBC, and 2.7% had triple-negative mBC. Most (90.9%) received at least one type of systemic treatment, with 16.2% undergoing chemotherapy, 61.3% anti-hormonal treatment, and 66.6% targeted cancer treatment as mono- or combination therapy.

**Table 2:** Sociodemographic and clinical characteristics (*n* = 111).

	Frequency (*n*)	Percent (%)
**Age**		
(Mean: 64.3 years. Range 37-88 years)		
< 50 years	13	11.7
50–60 years	33	29.7
61–70 years	30	27
71–80 years	27	24.3
> 80 years	8	7.2
**Years since first diagnosis** (Mean: 11.3 years)		
< 5 years	31	27.9
5–10 years	28	25.2
11–20 years	36	32.4
> 20 years	16	14.4
**Civil status**		
In a romantic relationship	58	52.3
Single	53	47.7
**Children**		
Yes	82	73.9
No	29	26.1
**Living situation**		
Living alone	46	41.4
Living with partner	38	34.2
Living with partner and children	17	15.3
Living with children	8	7.2
Other	2	1.8
**Occupational status**		
Full time (37 h/week)	17	15.3
Part time (15–37 h/week)	15	13.5
Part time (1–14 h/week)	8	7.2
On sick leave	7	6.3
Retired	59	53.2
Other	5	4.5
**Estrogen-receptor positive**		
Yes	99	89.2
No	12	10.8
**HER2-receptor positive**		
Yes	27	24.3
No	84	75.7
**Triple-negative**		
Yes	3	2.7
No	108	97.3
**Previous radiation therapy**		
Yes	82	73.9
No	29	26.1
**Previous surgery**		
Yes	94	84.7
No	17	15.3
**Previous systemic therapy**		
Yes	96	86.5
No	15	13.5
**Types of previous systemic therapy**		
Antihormonal therapy		
Yes	72	64.9
No	39	35.1
Chemotherapy		
Yes	63	56.8
No	47	42.3
Targeted therapy		
Yes	45	40.5
No	66	59.5
**Present systemic therapy**		
Yes	101	90.9
No	10	9.1
**Types of present systemic therapy**		
Antihormonal therapy		
Yes	68	61.3
No	43	38.7
Chemotherapy		
Yes	18	16.2
No	93	83.8
Targeted therapy		
Yes	74	66.6
No	37	33.3
**Present treatment line**		
First	55	49.5
Second	32	28.8
Third	14	12.6
Fourth	6	5.4
Fifth	4	3.6

### Quality of life

Table 3 gives an overview of QLQ-C30 and QLQ-BR45 scores for function- and symptom scales. Notably, the floor effect for symptom scales in the BR45 is 0% for 4 out of 7 symptom scales, reflecting that the null of the study population had the lowest score for this symptom. In general, the included women scored better in QoL on the QLQ-C30 than on the disease-specific QLQ-BR45.

**Table 3:** Quality of life among the study population using the cancer specific questionnaire. QLQ-C30* and QLQ-BR45**

	Median (IQR)	Floor effect (%)	Ceiling effect (%)	*n*
Quality of life among the study population using the cancer specific questionnaire. QLQ-C30*				
Overall quality of life	66.7 (33.3)	0.0	4.5	111
**Function scales**				
Physical function	80.0 (26.6)	0.0	20.7	111
Role function	83.3 (50.0)	4.5	37.8	111
Emotional function	83.3 (25)	0.0	19.8	111
Cognitive function	83.3 (33.3)	1.8	29.7	111
Social function	83.3 (33.3)	3.6	41.4	111
**Symptom scales**				
Fatigue	33.3 (33.4)	10.8	4.5	111
Nausea and vomiting	0.0 (16.7)	64.0	0.0	111
Pain	16.7 (41.7)	34.2	4.5	111
Shortness of breath	0.0 (33.3)	52.3	1.8	111
Insomnia	33.3 (66.4)	30.6	11.7	111
Appetite loss	0.0 (33.3)	64.9	1.8	111
Constipation	0.0 (33.3)	62.2	2.7	111
Diarrhoea	0.0 (33.3)	52.2	4.5	111
Financial difficulties	0.0 (16.7)	74.7	2.7	111
**QLQ-C30 sum-score**	79.3 (21.5)	0.0	1.8	111
Quality of life among the study population using the breast cancer specific module. QLQ-BR45**				
**Function scales**				
Body image	83.3 (33.3)	0.9	26.4	110
Future perspective	66.7 (33.4)	18.2	11.8	110
Sexual functioning	83.3 (33.3)	2.2	42.2	45
Sexual enjoyment	100.0 (33.3)	2.2	53.3	45
Breast satisfaction	33.3 (66.7)	35.5	18.2	110
**Symptom scales**				
Systemic therapy side effects	76.2 (19)	0.0	0.9	110
Upset by hair loss	100.0 (33.3)	6.4	58.7	109
Arm symptoms	88.9 (33.3)	1.8	35.5	110
Breast symptoms	100.0 (16.7)	0.0	53.6	110
Endocrine therapy symptoms	76.7 (20)	0.0	3.6	110
Skin mucosis symptoms	86.1 (22.2)	0.0	20.0	110
Endocrine sexual symptoms	76.7 (20)	0.9	42.7	110

IQR: interquartile range

* A high score for a function scale represents a high/healthy level of functioning. while a high score of a symptom scale/item represents a high level of symptomatology/problems

** A high score for a function scale represents a high/healthy level of functioning. however. the functional scales for Sexual Functioning. Sexual Enjoyment. and Breast Satisfaction are reversed. meaning that a higher score indicates lower functioning. A high score of a symptom scale/item represents a high level of symptomatology/problems

Quality of life measured using the QLQ-C30 sum-scores were highest with mono-therapy anti-hormonal treatment (80.7, IQR: 17.6), followed by targeted cancer treatment (78.8, IQR: 18.4), and lowest with chemotherapy (77.1, IQR: 24.8) (Table 4). Quality of life decreased with subsequent treatment lines (first line: 80.3, IQR: 20.7, fourth line: 67.4, IQR: 11.3). Women on sick leave showed poorer QoL than other occupational groups.

**Table 4:** Quality of life associated with sociodemographic characteristics for the study population based on the QLQ-C30 sum-score (n = 111).

	QLQ-C30 sum-score Median (IQR)
**Age**	
< 50 years	71.6 (13.2)
50–60 years	80.1 (19.5)
61–70 years	80.8 (23.9)
71–80 years	80.3 (20.5)
> 80 years	71.4 (23.7)
**Years since cancer**	
< 5 years	76.5 (9.3)
5–10 years	80.5 (20)
11–20 years	77.3 (16.4)
> 20 years	87.8 (12.7)
**Civil status**	
In a romantic relationship	79.0 (23.8)
Single	79.3 (18.7)
**Children**	
Yes	79.3 (21.5)
No	80.1 (21.7)
**Living situation**	
Living alone	79.4 (9.4)
Living with partner	80.8 (21.9)
Living with partner and children	71.2 (23.6)
Living with children	77.9 (10.2)
Other	-
**Occupational status**	
Full time (37 h/week)	85.4 (17.7)
Part time (1–14 h/week)	81.2 (9.3)
Part time (15–37 h/week)	73.1 (13.5)
On sick leave	67.4 (22.6)
Retired	79.2 (23.6)
Other	-
**Estrogen-receptor positive**	
Yes	79.3 (8.5)
No	78.6 (35.7)
**HER2-receptor positive**	
Yes	75.1 (18.8)
No	80.5 (22)
**Previous systemic therapy**	
Antihormonal therapy	
Yes	79.3 (21.4)
No	80.1 (22.6
Chemotherapy	
Yes	77.4 (7)
No	81.4 (24.3)
Targeted therapy	
Yes	73.6 (20.4)
No	81.3 (19.5)
**Present systemic therapy**	
Antihormonal therapy	
Yes	80.7 (17.6)
No	76.6 (24.2)
Chemotherapy	
Yes	77.1 (24.8)
No	79.5 (21.3)
Targeted therapy	
Yes	78.8 (18.4)
No	81.6 (27.8)
**Present treatment line**	
First	80.3 (20.7)
Second	82.9 (17.9)
Third	75.6 (19.6)
Fourth	67.4 (11.3)
Fifth	71.8 (21.6)

IQR: interquartile range

A high QLQ-C30 sum-score represents high quality of life

A trend, but no significant differences were seen between treatment lines and QoL measured using the QLQ-C30, but significant differences were seen for treatment lines and sexual enjoyment and upset by hair loss measured using the BR45. Women in early treatment lines 1 and 2 were more upset by hair loss than women in lines 3 to 5 (Tables 5 and 6).

**Table 5:** Odds for scoring under the median at a single QLQ-C30 scale among women in the 3–5-line therapy vs. women in the 1-2-line therapy.

	Below the median (*n*)	Above the median (*n*)	OR [95%CI] 1.00 (Ref.)	*P*-value*
**Overall QoL**				
1–2-line therapy	34	53	1.84	0.2
3–5-line therapy	13	11	[0.74;4.58]	
**Function scales**				
Physical function				
1–2-line therapy	36	51	1.20	0.7
3–5-line therapy	11	13	[0.48; 2.9]	
Role function				
1–2-line therapy	42	45	1.27	0.6
3–5-line therapy	13	11	[0.52; 3.13]	
Emotional function				
1–2-line therapy	36	51	1.67	0.3
3–5-line therapy	13	11	[0.67; 4.16]	
Cognitive function				
1–2-line therapy	34	53	1.84	0.2
3–5-line therapy	13	11	[0.74; 4.58]	
Social function				
1–2-line therapy	33	54	1.38	0.5
3–5-line therapy	11	13	[0.56; 3.45]	
**Symptom scales**				
Fatigue				
1–2-line therapy	26	51	0.78	0.6
3–5-line therapy	6	18	[0.28; 2.19]	
Pain				
1–2-line therapy	31	56	0.74	0.6
3–5-line therapy	7	17	[0.28; 1.99]	
Insomnia				
1–2-line therapy	28	59	0.70	0.5
3–5-line therapy	6	18	[0.25; 1.96]	
**Sum-score**				
1–2-line therapy	39	48	2.05	0.2
3–5-line therapy	15	9	[0.81; 5.19]	

* Significant *p* < 0.05.

For overall QoL. function scales and QLQ sum-score: OR above 1 indicates worse level of functioning/quality of life. OR below 1 indicates a better functioning/quality of life. For symptom scales: OR above 1 indicated lower symptomatology. OR below 1 indicated higher symptomatology.

**Table 6:** Odds for scoring under the median at a single QLQ-BR45 scale among women in the 3–5-line therapy vs. women in the 1–2-line therapy.

	Below the median (*n*)	Above the median (*n*)	OR [95% CI]1.00 (Ref.)	*P*-value*
**Function scales**				
Body image				
1–2-line therapy	10	49	1.85	0.2
3–5-line therapy	14	10	[0.74; 4.64]	
Future perspective				
1–2-line therapy	49	47	1.69	0.3
3–5-line therapy	14	10	[0.68; 4.22]	
Sexual functioning				
1–2-line therapy	14	21	3.50	0.1
3–5-line therapy	7	3	[0.77; 15.88]	
Sexual enjoyment				
1–2-line therapy	13	22	6.77	0.03*
3–5-line therapy	8	2	[1.24; 36.85]	
Breast satisfaction				
1–2-line therapy	36	50	0.99	0.9
3–5-line therapy	10	14	[0.40; 2.48]	
**Symptom scales**				
Systemic therapy side effects				
1–2-line therapy	40	46	0.97	0.9
3–5-line therapy	11	13	[0.39; 2.41]	
Upset by hair loss				
1–2-line therapy	41	44	0.21	< 0.01*
3–5-line therapy	20	4	[0.07; 0.68]	
Arm symptoms				
1–2-line therapy	33	53	2.25	0.08
3–5-line therapy	14	10	[0.90; 5.65]	
Breast symptoms				
1–2-line therapy	43	43	0.50	0.2
3–5-line therapy	8	16	[0.19; 1.29]	
Endocrine therapy symptoms				
1–2-line therapy	31	55	2.48	0.05
3–5-line therapy	14	10	[0.99; 6.25]	
Skin mucosis symptoms				
1–2-line therapy	42	44	1.24	0.6
3–5-line therapy	13	11	[0.50; 3.07]	
Endocrine sexual symptoms				
1–2-line therapy	45	41	1.52	0.4
3–5-line therapy	15	9	[0.60; 3.84]	

* Significant *p* < 0.05.

For function scales: OR above 1 indicates worse level of functioning/quality of life. OR below 1 indicates a better functioning/quality of life. For symptom scales: OR above 1 indicated lower symptomatology. OR below 1 indicated higher symptomatology.

Comparing women receiving monotherapy with targeted drugs (*n* = 15) to those receiving anti-hormonal or chemotherapy (*n* = 27), no significant differences were found in the OR between women in anti-hormonal therapy/chemotherapy and targeted monotherapy (Table 7); neither in the functional nor the symptom QLQ-C30-scales (Table 8 and 9), except for a border, significant difference was seen for insomnia in favour of targeted monotherapy.

**Table 7:** Quality of life scores stratified by treatment group

	Targeted therapy (*n* = 15)	Antihormonal or chemotherapy (*n* = 27)
QLQ-C30*	Median (IQR)	Median (IQR)
Overall quality of life	58.3 (29.2)	66.7 (25.0)
**Function scales**		
Physical function	73.3 (20.0)	80.0 (40.0)
Role function	83.3 (66.7)	100.0 (50.0)
Emotional function	83.3 (37.5)	83.3 (25.0)
Cognitive function	66.7 (33.3)	83.3 (33.3)
Social function	66.7 (33.4)	83.33 (33.3)
**Symptom scales**		
Fatigue	44.4 (22.3)	44.4 (38.9)
Nausea and vomiting	16.67 (33.3)	0.0 (16.7)
Pain	16.7 (41.7)	16.7 (41.7)
Shortness of breath	0.0 (33.3)	33.3 (33.3)
Insomnia	0.0 (33.3)	33.3 (66.7)
Appetite loss	33.3 (33.3)	0.0 (33.3)
Constipation	0.0 (33.3)	0.0 (33.3)
Diarrhoea	33.3 (33.3)	0.0 (33.3)
Financial difficulties	0.0 (50.0)	0.0 (0.0)
**QLQ-C30 sum-score**	73.6 (19.9)	81.6 (22.0)

QLQ-BR45**	Median (IQR)	Median (IQR)

**Function scales**		
Body image	75 (33.4)	83.3 (33.3)
Future perspective	66.7 (33.4)	66.7 (33.4)
Sexual functioning	75.0 (16.6)	75 (33.3)
Sexual enjoyment	83.3 (33.3)	83.3 (33.3)
Breast satisfaction	33.3 (83.3)	33.3 (91.7)
**Symptom scales**		
Systemic therapy side effects	80.9 (28.6)	80.9 (14.3)
Upset by hair loss	100.0 (100.0)	100.0 (33.3)
Arm symptoms	88.9 (16.7)	77.8 (33.3)
Breast symptoms	100.0 (20.8)	100.0 (8.3)
Endocrine therapy symptoms	80.0 (20.0)	76.7 (18.4)
Skin mucosis symptoms	72.2 (41.6)	83.3 (16.7)
Endocrine sexual symptoms	83.3 (20.8)	100.0 (20.8)

IQR: interquartile range

* A high score for a function scale represents a high/healthy level of functioning. while a high score of a symptom scale/item represents a high level of symptomatology/problems

** A high score for a function scale represents a high/healthy level of functioning. however. the functional scales for Sexual Functioning. Sexual Enjoyment. and Breast Satisfaction are reversed. meaning that a higher score indicates lower functioning. A high score of a symptom scale/item represents a high level of symptomatology/problems

**Table 8:** Odds for scoring under the median at a single QLQ-C30 scale among women receiving targeted monotherapy vs. antihormonal or chemotherapy.

	Below the median (*n*)	Above the median (*n*)	OR [95% CI] 1.00 (Ref.)	*P*-value*
**Overall quality of life**				
Antihormonal/chemotherapy	12	15	1.43	0.6
Targeted monotherapy	8	7	[0.40; 5.07]	
**Function scale**				
Physical function				
Antihormonal/chemotherapy	10	17	1.94	0.3
Targeted monotherapy	8	7	[0.54; 6.99]	
Role function				
Antihormonal/chemotherapy	12	15	1.09	0.9
Targeted monotherapy	7	8	[0.31; 3.88]	
Emotional function				
Antihormonal/chemotherapy	11	16	1.27	0.7
Targeted monotherapy	7	8	[0.36; 4.54]	
Cognitive function				
Antihormonal/chemotherapy	11	16	1.66	0.4
Targeted monotherapy	8	7	[0.47; 5.93]	
Social function				
Antihormonal/chemotherapy	9	18	3.00	0.09
Targeted monotherapy	9	6	[0.81; 11.08]	
**Symptom scale**				
Fatigue				
Antihormonal/chemotherapy	9	18	0.31	0.2
Targeted monotherapy	2	13	[0.06; 1.67]	
Pain				
Antihormonal/chemotherapy	10	17	0.85	0.8
Targeted monotherapy	5	10	[0.23; 3.21]	
Insomnia				
Antihormonal/chemotherapy	8	19	3.56	0.06
Targeted monotherapy	9	6	[0.95; 13.37]	
**QLQ-C30 sum-score**				
Antihormonal/chemotherapy	12	15	1.88	0.3
Targeted monotherapy	9	6	[0.52; 6.76]	

* Significant *p* < 0.05.

For function scales: OR above 1 indicates worse level of functioning/quality of life. OR below 1 indicates a better functioning/quality of life.

For symptom scales: OR above 1 indicated lower symptomatology. OR below 1 indicated higher symptomatology.

**Table 9:** Odds for scoring under the median at a single QLQ-BR45 scale among women receiving targeted monotherapy vs. antihormonal or chemotherapy.

	Below the median (*n*)	Above the median (*n*)	OR [95% CI]1.00 (Ref.)	*P*-value*
**Function scale**				
Body image				
Antihormonal/chemotherapy	13	14	1.23	0.8
Targeted monotherapy	8	7	[0.35; 4.36]	
Future perspective				
Antihormonal/chemotherapy	13	14	0.94	0.9
Targeted monotherapy	7	8	[0.27; 3.34]	
Sexual functioning				
Antihormonal/chemotherapy Targeted monotherapy	5 3	5 3	1.00 [0.13; 7.57]	1.0
Sexual enjoyment				
Antihormonal/chemotherapy	5	5	2.00	0.5
Targeted monotherapy	4	2	[0.24; 16.36]	
Breast satisfaction				
Antihormonal/chemotherapy	10	17	1.13	0.8
Targeted monotherapy	6	9	[0.31; 4.14]	
**Symptom scale**				
Systemic therapy side effects				
Antihormonal/chemotherapy	10	17	1.13	0.8
Targeted monotherapy	6	9	[0.31; 4.14]	
Upset by hair loss				
Antihormonal/chemotherapy	9	18	0.50	0.4
Targeted monotherapy	3	12	[0.11; 2.23]	
Arm symptoms				
Antihormonal/chemotherapy	14	13	0.34	0.1
Targeted monotherapy	4	11	[0.09; 1.33]	
Breast symptoms				
Antihormonal/chemotherapy	11	16	1.27	0.7
Targeted monotherapy	7	8	[0.36; 4.54]	
Endocrine therapy symptoms				
Antihormonal/chemotherapy	12	15	0.63	0.5
Targeted monotherapy	5	10	[0.17; 2.33]	
Skin mucosis symptoms				
Antihormonal/chemotherapy	16	11	1.03	0.9
Targeted monotherapy	9	6	[0.28; 3.74]	
Endocrine sexual symptoms				
Antihormonal/chemotherapy	11	16	2.91	0.1
Targeted monotherapy	10	5	[0.78; 10.89]	

* Significant *p* < 0.05.

For function scales: OR above 1 indicates worse level of functioning/quality of life. OR below 1 indicates a better functioning/quality of life.

For symptom scales: OR above 1 indicated lower symptomatology. OR below 1 indicated higher symptomatology.

**Figure 2:**
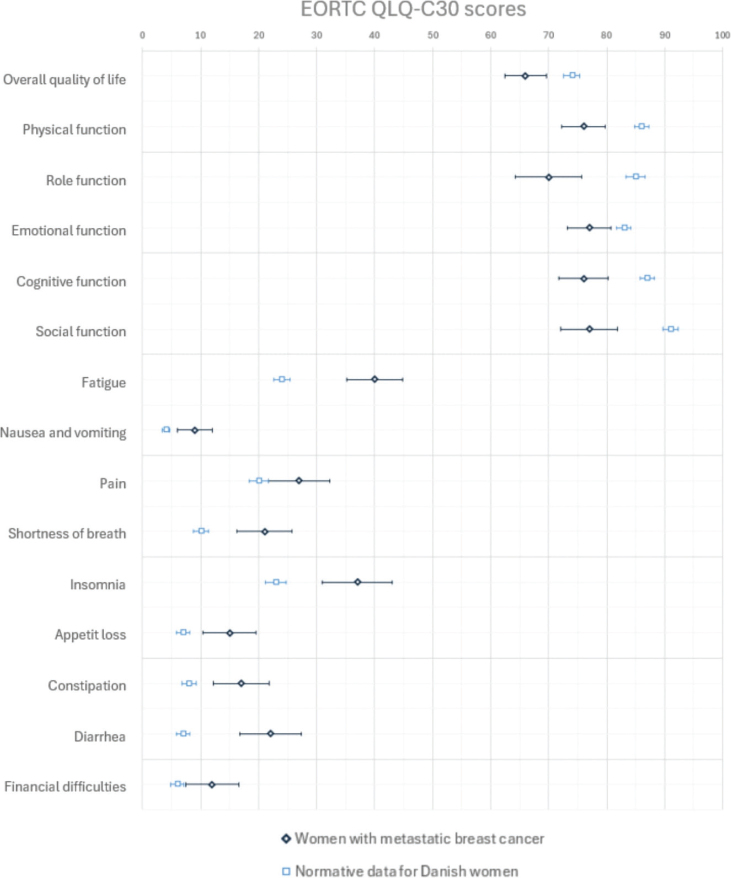
Visual comparison of normative data for EORTC QLQ-C30 in a Danish female population given as means with 95% CI and QLQ-C30 for the present mBC population (Mean, 95% CI).

In a visual graphically comparison (Figure 2), women with mBC had poorer function and higher symptom burdens than EORTC QLQ-C30 normative data.

## Discussion

As the palliative oncological treatments improve, the number of patients living with a cancer disease increase; this population can be referred to as metastatic survivors. Women living with mBC, mBC survivors, have often undergone several treatment lines and can suffer from accumulated toxicities. Our results show that patients with 1–2 treatment lines have lower QoL related to sexual enjoyment and were upset by hair loss measured in BR45 than patients with three or more treatment lines. No significant differences in QoL scores were found between participants receiving anti-hormonal therapy/chemotherapy, and those on targeted monotherapy. Despite the surprising fact that only a border significant difference in insomnia was found between the two groups, the authors acknowledge that a significant difference in symptoms was challenging to detect due to the relatively small sample size. These findings suggest accumulated toxicity and impaired QoL in mBC independent of current specific treatment regimens, which need further attention in research as well as in the cancer clinic [8].

The observed differences in QoL with successive lines of treatment are reflected in previous studies that have reported poorer QoL in women with metastasis compared to local disease [11], and they are likely associated with cumulative treatment load and late effects of treatments. Previous randomised clinical trials comparing treatment with the immune-check-inhibitor Pembrolizumab versus chemotherapy showed that patients receiving Pembrolizumab had better QoL than those receiving chemotherapy [7]. However, in line with our findings, Tommasi et al. conclude in a literature review that toxicity seems to accumulate from the time of breast cancer diagnosis, and it is important to try to reduce or even prevent the onset of adverse events related to breast cancer treatments that can afflict and worsen QoL [28]. Although introduction of less toxic drugs such as Pembrolizumab seems promising, side effects from such will be added to existing symptoms when new drugs are introduced in a treatment trajectory.

Our study is hampered by the fact that all targeted therapies have been pooled, including, for example atezolizumab and palbociclib in the targeted therapy, and likewise, for chemotherapy pooling, for example gemcitabine and docetaxel, although these drugs express different toxicities. This was done because introducing modern targeted treatments with, for example small molecules promised less toxicity with a good toxicity profile and less severe toxicity [29]. Further, the pooling of antihormone therapy and chemotherapy might hide the presumable less toxic profile of antihormone when given alone. To some extent, the hypothesis of less toxicity in more accurate treatment seems to account for breast cancer treatment. Also, for example anti-HER2-treatment, Sodergren et al. have shown a very low frequency of toxicities, but some very severe, such as cardiac problems, when reviewing the literature [30]. Our data, arising from the exploration of a relatively limited mBC population, however, suggest that it is important to bear in mind not only the toxicity of the ongoing treatment but the cumulated experience and toxicity from a lived life with breast cancer.

In our study, approximately 10% of the study population were not treated with therapy at the time of survey response. If this group had been larger, a comparison with women in treatment would have been interesting to investigate a potential difference in symptoms between these groups. These women could represent participants included right before the start of systemic treatment.

## Strength and limitations

A strength of this study is that we included patients with mBC across all stages independently of treatment line or type, and this allowed us to compare across treatment lines. However, the study is limited by its cross-sectional nature which prevents us from making causal interpretations. Also, the relatively small population allowed limited statistical power as well as representativeness of all women with metastatic breast cancer. It is a limitation that all targeted therapies have been pooled and compared to chemotherapy. This was, however, necessary to create meaningful sample sizes (15 vs. 27), which at the same time are relatively small. Therefore, results should be interpreted with caution.

Regarding the choice of statistical measures and analyses, efforts were made to adapt the analytical methods to the data distribution. The data did not follow a normal distribution on most scales in the QLQ-C30 and QLQ-BR45, which is typical for data from QoL questionnaires [31]. Based on this, we presented medians rather than means in the descriptive analyses. This decision was made even though most identified studies investigating the QoL using the QLQ-C30 present mean values [11, 32–35]. A drawback of this approach is that it limits direct comparison of the figures, although it is still possible to observe trends. Another disadvantage of presenting medians is that they may obscure nuances if there is significant variation within individual categories or groups. However, the advantage of presenting medians is that extreme values influence them less compared to means. This was relevant in this study as the data had fluctuations and outliers.

Further research is needed to elucidate the QoL-landscape of mBC at diagnosis and during the cancer trajectory, including those who live with mBC for many years, an under-researched need not only in breast cancer but also in other malignant entities, where the number of metastatic survivors is increasing [36].

## Perspectives

The increasing population of women living long-term with mBC calls for increased attention from healthcare professionals on accumulated toxicity [28]. At present, treatment with a limited toxicity profile can be just as burdensome to the patient as a prior, more toxic treatment, which has left the patient with long-time chronic side effects persisting throughout new, for example modern targeted therapies. It is, therefore, important to develop models of care for patients undergoing long-term treatment that focus on identifying and managing the many acute and chronic symptoms to help them maintain QoL for as long as possible.

International guidelines have recently been developed to support metastatic cancer patients [37]. Few supportive care interventions have been explicitly addressing the needs of patients with metastatic cancer. An exemption is the manualised psychotherapeutic intervention Managing Cancer and Living Meaningfully (CALM), which was specifically adapted for patients with advanced cancer and has shown effect in reducing symptoms of depression [38]. Our results also show fatigue and insomnia to be among the symptom burdens experienced by women with mBC compared to general population norms. Current guidelines for treating fatigue and insomnia recommend cognitive behavioural therapy and physical exercise, but the evidence has primarily been based on studies done in cured cancer survivors [39, 40]. Future studies are needed to develop interventions that are tailored to metastatic cancer survivors.

In order to support patients who may live for many years with mBC and with more extended periods of stability, new eHealth approaches such as smartphone applications enabling delivery of patient education and collection of patient-reported outcomes, among others, may be relevant to further target the mBC population.

Our findings point to the need for exploring metastatic survivorship in breast cancer in more detail on a larger scale, focussing on stratifying into different cancer treatment groups to obtain knowledge that could be used in guidelines for supporting women with mBC.

## Conclusion

In women with mBC, QoL differs with successive lines of treatment. We found no significant differences between patients receiving anti-hormonal therapy/chemotherapy, and those on targeted monotherapy. No overlap with normative data was seen for all functional scales and symptom scales. The lack of significant variance between treatment groups could be attributed to increased toxicity across successive treatment lines, which must be acknowledged in the growing group of mBC survivors.

## Data Availability

The datasets from the current study are available upon reasonable request by contacting the corresponding author.
